# A Validated Composite Score Demonstrates Potential Superiority to MELD-Based Systems in Predicting Short-Term Survival in Patients with Liver Cirrhosis and Spontaneous Bacterial Peritonitis—A Preliminary Study

**DOI:** 10.3390/diagnostics13152578

**Published:** 2023-08-02

**Authors:** Yan-Ting Lin, Wei-Ting Chen, Tsung-Han Wu, Yu Liu, Li-Tong Liu, Wei Teng, Yi-Chung Hsieh, Yen-Mu Wu, Chien-Hao Huang, Chao-Wei Hsu, Rong-Nan Chien

**Affiliations:** 1 Department of Hepatology, Department of Gastroenterology and Hepatology, Chang-Gung Memorial Hospital, Linkou Medical Center, Taoyuan 33305, Taiwanliu870223@gmail.com (Y.L.); nana2052727@gmail.com (L.-T.L.); cutebuw@yahoo.com.tw (Y.-C.H.);; 2 College of Medicine, Chang-Gung University, Taoyuan 33302, Taiwan; 3 Department of General Surgery, Chang-Gung Memorial Hospital, Linkou Medical Center, Taoyuan 333, Taiwan; 4 Department of Infectious Disease, Chang-Gung Memorial Hospital, Linkou Medical Center, Taoyuan 33305, Taiwan; yenmuwu@gmail.com

**Keywords:** spontaneous bacterial peritonitis, liver cirrhosis, short-term mortality, MELD-based prediction models, sepsis, hepatorenal syndrome, under receiver operating characteristic curve (AUROC), 5-fold cross internal validation, subgroup sensitivity analysis

## Abstract

Background: Spontaneous bacterial peritonitis (SBP) is a severe complication in cirrhosis patients with ascites, leading to high mortality rates if not promptly treated. However, specific prediction models for SBP are lacking. Aims: This study aimed to compare commonly used cirrhotic prediction models (CTP score, MELD, MELD-Na, iMELD, and MELD 3.0) for short-term mortality prediction and develop a novel model to improve mortality prediction. Methods: Patients with the first episode of SBP were included. Prognostic values for mortality were assessed using AUROC analysis. A novel prediction model was developed and validated. Results: In total, 327 SBP patients were analyzed, with HBV infection as the main etiologies. MELD 3.0 demonstrated the highest AUROC among the traditional models. The novel model, incorporating HRS, exhibited superior predictive accuracy for in-hospital in all patients and 3-month mortality in HBV-cirrhosis, with AUROC values of 0.827 and 0.813 respectively, surpassing 0.8. Conclusions: MELD 3.0 score outperformed the CTP score and showed a non-significant improvement compared to other MELD-based scores, while the novel SBP model demonstrated impressive accuracy. Internal validation and an HBV-related cirrhosis subgroup sensitivity analysis supported these findings, highlighting the need for a specific prognostic model for SBP and the importance of preventing HRS development to improve SBP prognosis.

## 1. Introduction

Patients with liver cirrhosis often exhibit an impaired defense against bacteria, leading to reduced bacterial clearance [1]. This immune defect, coupled with increased intestinal permeability and gut bacterial overgrowth, facilitates bacterial translocation. As a result of the pathological bacterial translocation in liver cirrhosis, the bacterial infection is either present on admission or develops during hospitalization in approximately 30% of patients with cirrhosis [2]. The most prevalent form of these infections is spontaneous bacterial peritonitis (SBP) [2].

SBP is characterized by the spontaneous infection of ascitic fluid, occurring in cirrhotic patients in the absence of an intra-abdominal source of infection [3]. The presence of SBP in patients with ascites can lead to high mortality rates if not promptly treated [4,5]. Additionally, the concurrence of hepatorenal syndrome (HRS), characterized by acute kidney injury in patients with acute or chronic liver disease and affecting up to 30% of patients with SBP, further increases the risk of mortality [3]. One-year overall mortality rates for SBP range from 53.9% to 78% [6,7], indicating that SBP may represent the final clinical stage of liver cirrhosis [8]. Given the serious consequences of SBP, accurate prediction of mortality is crucial for guiding clinical decisions and considering liver transplantation. However, it is worth noting that specific prediction models for SBP are currently lacking [5].

The model for end-stage liver disease (MELD) has demonstrated superior accuracy in predicting 3-month mortality compared to the Child–Turcotte–Pugh (CTP) score in cirrhotic patients awaiting liver transplantation in the United States [9]. Therefore, the MELD has been widely adopted by the United Network for Organ Sharing (UNOS) and other countries since 2002 to improve the prioritization of liver transplantation (LT) waiting lists [10]. Several MELD-based models, such as the model for end-stage liver disease with the incorporation of serum sodium (MELD-Na) [11] and the integrated model for end-stage liver disease (iMELD) score [12], have been developed to enhance mortality prediction in cirrhotic patients [13,14]. In recent years, MELD has been updated to version 3.0 to address the need for reducing deaths while patients are on the waitlist for liver transplantation [15]. Despite the good prognostic performance of iMELD and MELD-Na for 3-month and 6-month mortality in cirrhosis patients [16,17], their applicability in subgroups with liver cirrhosis-related complications, such as SBP, remains limited [18,19]. This limitation is particularly relevant in regions with a high prevalence of chronic hepatitis B, such as Taiwan, where chronic hepatitis B virus (HBV) infection is the leading cause of HCC, especially in Asia and Africa [20,21].

Currently, there is a lack of specific prediction models for SBP. However, in our previous study, we demonstrated that among the MELD-based models, iMELD and MELD-Na exhibited the highest area under the receiver operating characteristic curve (AUROC) and significantly outperformed other models in predicting 3-month and 6-month mortalities in patients with HBV-related liver cirrhosis and SBP [22]. Subsequently, we also identified that factors such as bacteremia (sepsis), hepatorenal syndrome (HRS), and serum creatinine levels had a stronger impact on short-term survival compared to types of SBP or microbial patterns in these patients [23]. Therefore, the objectives of our study are to (i) compare commonly used cirrhotic mortality models, including the recently updated MELD 3.0 [15], and (ii) explore the potential for developing a superior prediction model to accurately predict short-term mortality (in-hospital, 3 and 6 month) in patients with SBP.

## 2. Materials and Methods

### 2.1. Patient Selection

**Figure 1:**
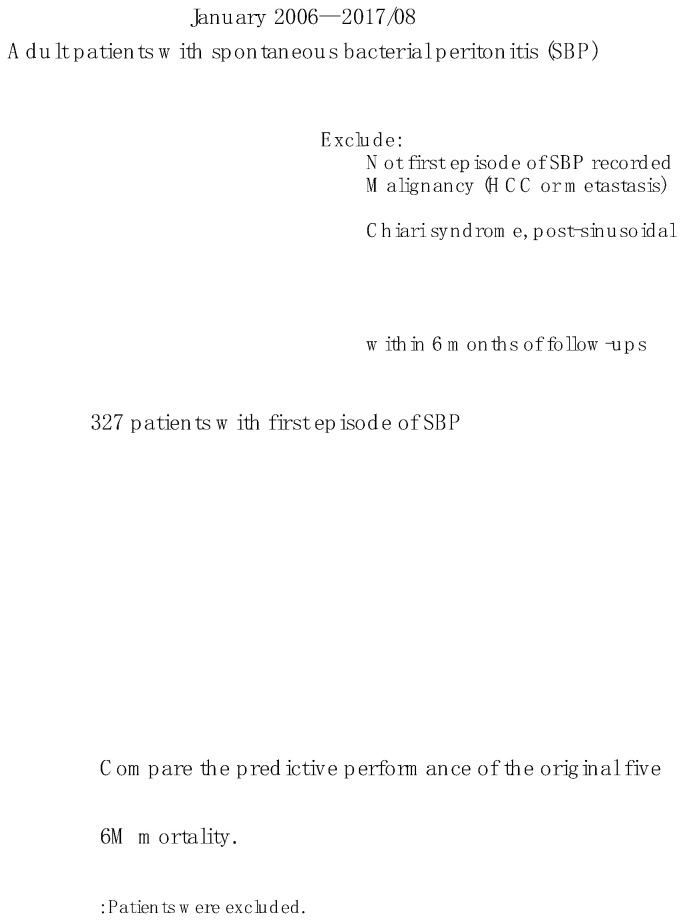
Flowchart of the study. Following the inclusion and exclusion criteria, a total of 327 patients diagnosed with the first episode of SBP were included in the study analysis.

A retrospective cohort study was conducted with approval from the ethical committees of Chang Gung Memorial Hospital. As shown in Figure 1, adult patients diagnosed with SBP and liver cirrhosis between January 2006 and August 2017 were included in the study. Patient data, including clinical, demographic, hematological, and biochemical information, were collected at the time of SBP diagnosis. Various scoring systems, such as the CTP score and the four MELD-based scores (MELD, MELD-Na, iMELD, MELD 3.0), were calculated. Patients were followed up until March 2018 or until death. Patients who had previous episodes of SBP, had malignancy, had ascites not related to cirrhosis, or underwent liver transplantation within 6 months of follow-ups were excluded from mortality calculations. Consequently, a total of 327 patients diagnosed with the first episode of SBP were enrolled in the analysis.

### 2.2. Diagnosis, Definition, and Management of Liver Cirrhosis with Ascites and Spontaneous Bacterial Peritonitis

The diagnosis of liver cirrhosis (LC) with ascites was based mainly on the following criteria: (1) LC diagnosed based on histopathology (liver biopsy) or compatible clinical features, laboratory data, sonographic/computed tomography/magnetic resonance imaging findings typical for liver cirrhosis [24], or non-invasive testing such as FibroScan [25]; (2) ascites caused by portal hypertension (serum-ascites albumin gradient (SAAG) ≥ 1.1 g/dL) [26]; (3) exclusion of other underlying disease that can cause ascites such as malignancy (HCC or metastasis), right-sided congestive heart failure/constrictive pericarditis, Budd–Chiari syndrome, post-sinusoidal obstruction syndrome, portal or splenic vein thrombosis, and the possibility of schistosomiasis. 

SBP is suspected in cirrhotic patients presenting with suggestive symptoms and signs, such as fever, abdominal pain, altered mental status, abdominal tenderness, or hypotension [27]. SBP is diagnosed upon an absolute neutrophil count in ascites fluid of ≥250 cells/mm^3^ and/or positive ascites culture, in the absence of a surgically treatable intra-abdominal source of infection and other causes of elevated ascites neutrophil count, such as hemorrhage, pancreatitis, peritoneal tuberculosis, or carcinomatosis [28]. Since infections are common in cirrhotic patients with variceal bleeding [29], paracentesis should be performed upon hospital admission in all cirrhotic patients with ascites, even if admitted for reasons other than ascites [30]. Baseline laboratory data were collected when SBP was suspected, and prompt diagnosis was facilitated by efficient laboratory services. In our facility, the results of ascites fluid routine can be obtained within 2 h, enabling a timely diagnosis of SBP within 1 day.

The management of SBP follows the recommendations of the International Ascites Club [31] and the AASLD/EASL/JAMA guidelines [5,25]. In the case of HBV-related cirrhotic patients, antiviral agents are administered if they meet the latest APASL guideline [32,33,34].

### 2.3. Primary Outcomes and Scheduled Follow-Up Periods

Due to the high short-term mortality associated with SBP, the primary outcome of this study was defined as in-hospital mortality, as well as mortality at 3 months (3 M) and 6 months (6 M) following diagnosis. Each patient was followed up at least every 3 to 6 months until the date of death or March 2018, whichever occurred first. 

### 2.4. Calculation of the Child–Turcotte–Pugh, MELD, MELD-Na, iMELD, and MELD 3.0 Scores

The Child–Turcotte–Pugh (CTP) score was calculated according to the established criteria [35]. MELD, MELD-Na, iMELD, and MELD 3.0 scores were measured using the formula proposed in previous studies. The MELD score was 11.2 × loge (INR) + 9.57 × loge (creatinine, mg/dL) + 3.78 × loge (bilirubin, mg/d) + 6.43), with a lower bound of 1 for all 3 variables and an upper bound of 4 for serum creatinine [9]. The MELD-Na score was MELD-Na − [0.025 × MELD × (140 − Na)] + 140, with Na was bounded at 125 and 140 [11]. The iMELD score was MELD + (Age × 0.3) − (0.7 × Na + 100) [12]. The MELD 3.0 was 1.33 × (Female) + 4.56 × ln (Serum bilirubin) + 0.82 × (137 − Sodium) − 0.24 × (137 − Sodium) × ln (Serum bilirubin) + 9.09 × ln (INR) + 11.14 × ln (Serum creatinine) + 1.85 × (3.5 − Serum albumin) − 1.83 × (3.5 − Serum albumin) × ln (Serum creatinine) + 6, rounded to the nearest integer [15].

### 2.5. Statistical Analysis 

The data were properly tabulated for statistical analysis. Normally distributed continuous data were presented as mean ± standard deviation (SD) and compared using an independent Student’s *t*-test. Non-normally distributed continuous data were expressed as median and interquartile range (IQR) and compared using the Mann–Whitney U test. Categorical variables were presented as frequencies and percentages and compared using the χ^2^ test or Fisher’s exact test. The predictive ability of the novel model was evaluated by calculating the area under the receiver operating characteristic curve (AUROC) and compared to existing scoring systems such as CTP, MELD, MELD-Na, iMELD, and MELD 3.0. The predictive performance of each scoring system in predicting mortality was compared using the DeLong test. The best cut-off values for the new SBP scoring system were determined using the Youden Index. All statistical analyses were performed using IBM SPSS Statistics version 25 (SPSS Inc., Chicago, IL, USA) and R 4.2.2 (R Core Team, 2021; Vienna, Austria). A *p*-value less than 0.05 was considered statistically significant.

### 2.6. Construction of a Novel Prognostic Model

Using the current dataset, the primary outcomes were the dependent variable in the construction of a novel prognostic model. Initially, several independent variables were included, such as patients’ demographics, etiology of cirrhosis, ascites PMN, the presence or absence of sepsis, ascites, hepatic encephalopathy (HE), HRS, serum Cr, Na, K, total bilirubin, albumin, INR, hemoglobin, WBC, and PLT. Variables with a *p* < 0.20 in the univariate analysis were included in a multivariate logistic regression analysis [36] to identify independent predictors that could predict primary outcomes with an AUROC > 0.8, indicating a good discriminatory ability [37].

### 2.7. Model Validation and Sensitivity Analysis

For model validation, internal validation was performed using 5-fold cross-validation, which involved dividing a group of 327 patients into 5 equal subsets. The model was trained 5 times, with each iteration using 4 out of the 5 subsets as the training data (261 patients), while the remaining subset (66 patients) served as the test (validation) set. The performance of the model was assessed using various metrics, including AUROC, Brier scores, and accuracy at intervals. The results of the internal validation, including AUROC, Brier scores, and accuracy, can be found in Supplementary Table S4 and Supplementary Figure S1. A subgroup sensitivity analysis was performed specifically on the HBV subgroup to further examine the performance of the predictive models.

## 3. Results

### 3.1. Patients’ Baseline Characteristics

After applying the inclusion and exclusion criteria, a total of 327 patients diagnosed with the first episode of SBP were enrolled in the study. Table 1 presents the demographic characteristics of the patients, etiology of cirrhosis, hemogram and biochemical laboratory data measured at diagnosis, as well as the scores of the CTP and four MELD-based models. The patients were predominantly middle-aged and predominantly male. Among the patients, 69.2% had chronic hepatitis virus infections, with HBV infection being the most common etiology. Among the HBV-infected patients, 57.4% (85/148) received antiviral treatment. Sepsis was diagnosed in 12.8% of the SBP patients based on positive blood culture and evidence of systemic inflammatory response syndrome. Hepatorenal syndrome was present in 9.5% of the patients, and 25.7% had HE grade ≥ 1.

Table 1 also displays the interpretation of various abnormal laboratory data in patients with SBP. These abnormalities include renal function impairment, hyperbilirubinemia, hyponatremia, hypoalbuminemia, prolonged INR for prothrombin time, anemia, and thrombocytopenia. The mean white blood cell counts (WBCs) in blood were within the normal range, while the ascites polymorphonuclear leukocytes (PMNs) counts were >250 cells/μL. The mean CTP class was C. The scores for the four MELD-based models were displayed in Table 1.

### 3.2. Comparison of Score Differences among CTP and Four MELD-Based Models between In-Hospital, 3-Month, and 6-Month Mortality and Non-Mortality Groups

Table 2 presents the statistical differences between the in-hospital, 3-month, and 6-month mortality groups and the non-mortality group in terms of CTP and four MELD-based model scores. The in-hospital mortality rate was 39.4%. The primary causes of in-hospital mortality were sepsis with multiple organ failures (81 patients, 62.8%), end-stage liver disease (ESLD)-related multiple organ failures (28 patients, 21.7%), massive gastrointestinal bleeding with shock (13 patients, 10.1%), and other causes (7 patients, 5.4%).

The cumulative 3-month and 6-month mortality rates were 51.4% and 60.9%, respectively. Significant differences were observed between the in-hospital, 3-month, and 6-month mortality and non-mortality groups in terms of the CTP score and the four MELD-based model scores (Table 2).

### 3.3. Comparison of the Predicting Performance of the Original CTP, MELD, MELD-Na, iMELD, MELD 3.0, and the New SBP Scores for In-Hospital, 3-Month, and 6-Month Mortality Prediction Using AUROC and DeLong Test

To compare the predictive ability of the CTP score and the four MELD-based model scores for short-term mortality prediction (in-hospital, 3-month, and 6-month mortality), the AUROC was calculated for each score and compared. 

**Figure 2:**
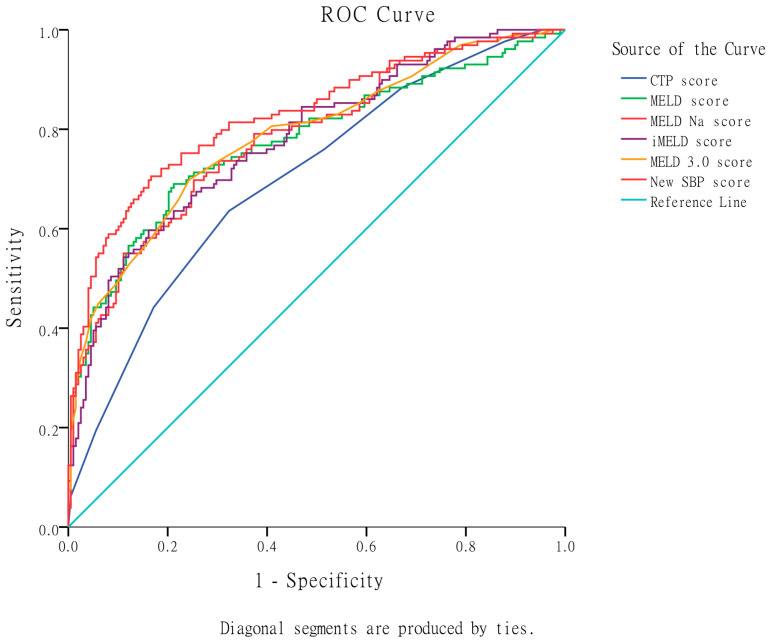
Comparison of the original CTP, MELD, MELD-Na, iMELD, MELD 3.0, and the new SBP scores in predicting in-hospital mortality based on AUROC. The MELD 3.0 score showed significant superiority over the CTP score and non-significant improvement over MELD, MELD-Na, and iMELD scores. The newly developed SBP score exhibited an impressive AUROC of 0.827 and demonstrated significant superiority over the CTP score, MELD, MELD-Na, and iMELD scores. Furthermore, the new SBP score displayed a marginal advantage compared to the MELD 3.0 score.

In terms of predicting in-hospital mortality, as shown in Table 3 and Figure 2, the MELD 3.0 score exhibited the highest AUROC (0.786), followed by MELD-Na (0.783), iMELD (0.780), MELD (0.775), and CTP score (0.701). The MELD 3.0 score was significantly superior to the CTP score and non-significantly superior to MELD, MELD-Na, and iMELD scores in predicting in-hospital mortality.

**Figure 3:**
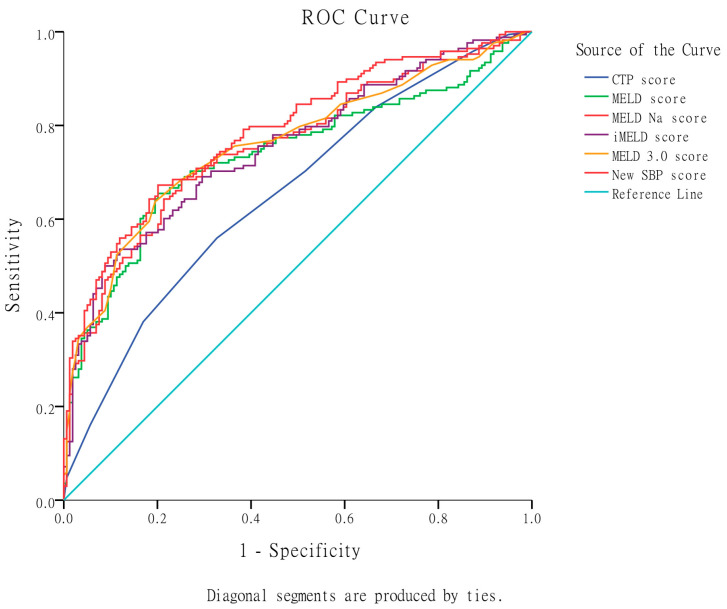
Comparison of the original CTP, MELD, MELD-Na, iMELD, MELD 3.0, and the new SBP scores in predicting 3-month mortality using AUROC. The MELD 3.0 score exhibited the highest AUROC, followed by MELD-Na, iMELD, MELD, and CTP scores. The MELD 3.0 score was significantly superior to the CTP score and MELD score, and non-significantly superior to MELD-Na and iMELD scores. The newly developed SBP score demonstrated significant superiority over the CTP score and MELD. However, it did not show significant superiority over the MELD-Na, iMELD, and MELD 3.0 scores.

In terms of predicting 3-month mortality, Table 4 and Figure 3 demonstrate that the MELD 3.0 score exhibited the highest AUROC (0.760), followed by MELD-Na (0.755), iMELD (0.753), MELD (0.737), and CTP score (0.656). The MELD 3.0 score was significantly superior to the CTP score and MELD score, and non-significantly superior to MELD-Na and iMELD scores in predicting 3-month mortality.

**Figure 4:**
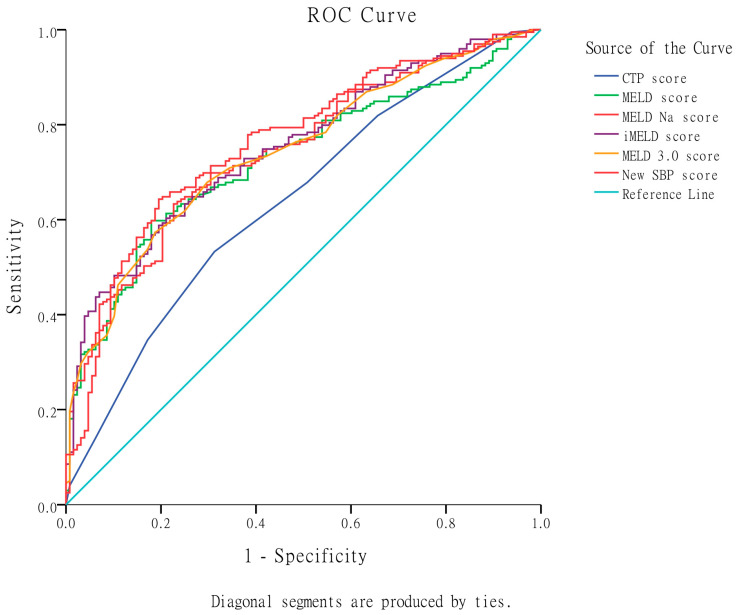
Comparison of the original CTP, MELD, MELD-Na, iMELD, MELD 3.0, and the new SBP scores in predicting 6-month mortality by AUROC. The iMELD score exhibited the highest AUROC, followed by MELD-Na, MELD 3.0, MELD, and CTP scores. The iMELD score was significantly superior to the CTP score and non-significantly superior to MELD-Na, MELD 3.0, and MELD scores. The newly developed SBP score displayed significant superiority over the CTP score. However, it did not show significant superiority over the MELD, MELD-Na, iMELD, and MELD 3.0 scores.

In terms of predicting 6-month mortality, Table 5 and Figure 4 demonstrate that the iMELD score exhibited the highest AUROC (0.752), followed by MELD-Na (0.745), MELD 3.0 (0.742), MELD (0.728), and CTP score (0.640). The iMELD score was significantly superior to the CTP score and non-significantly superior to MELD-Na, MELD 3.0, and MELD scores in predicting 6-month mortality.

### 3.4. Development of the New SBP Score Model

We developed a new SBP score model to enhance the accuracy of predicting in-hospital, 3-month, and 6-month mortalities. Based on the methods outlined in the Section 2, the new SBP score model was constructed by integrating age, serum creatinine, total bilirubin, International Normalized Ratio (INR), platelet count, as well as the presence of sepsis and hepatorenal syndrome (HRS). 

The formula of the new SBP score model is as follows: 

For in-hospital mortality: Score = −5.982 + 0.040 × Age + 0.202 × serum Cr + 0.117 × serum total bilirubin + 0.777 × INR + 0.004 × PLT + 1.009 × sepsis (0: without sepsis/1: with sepsis) + 1.576 × HRS (0: without HRS/1: with HRS).

For 3-month mortality: Score = −4.816 + 0.037 × Age + 0.132 × serum Cr + 0.136 × serum total bilirubin + 0.482 × INR + 0.005 × PLT + 0.978 × sepsis (0: without sepsis/1: with sepsis) + 1.542 × HRS (0: without HRS/1: with HRS).

For 6-month mortality: Score = −4.837 + 0.036 × Age + 0.604 × serum Cr + 0.112 × serum total bilirubin + 0.500 × INR + 0.005 × PLT + 0.661 × sepsis (0: without sepsis/1: with sepsis) + 0.858 × HRS (0: without HRS/1: with HRS).

### 3.5. Comparative Analysis of the Predictive Performance between the New SBP Score and the Original CTP, MELD, MELD-Na, iMELD, and MELD 3.0 Scores for In-Hospital, 3-Month, and 6-Month Mortality Prediction Utilizing AUROC and DeLong Test

According to the results presented in Table 3 and Figure 2, the newly developed SBP score model exhibited an impressive AUROC of 0.827. Notably, it demonstrated significant superiority over the CTP score, MELD, MELD-Na, and iMELD scores in predicting in-hospital mortality. Furthermore, the new SBP score model displayed a marginal advantage compared to the MELD 3.0 score in its predictive capability for in-hospital mortality.

Moreover, in terms of predicting 3-month mortality, the newly developed SBP score model demonstrated significant superiority over the CTP score and MELD (Table 4 and Figure 3). However, it did not show significant superiority over the MELD-Na, iMELD, and MELD 3.0 scores.

Additionally, when considering the prediction of 6-month mortality, the newly developed SBP score model displayed significant superiority over the CTP score (Table 5 and Figure 4). However, it did not show significant superiority over the MELD, MELD-Na, iMELD, and MELD 3.0 scores and its AUROC did not surpass 0.8.

### 3.6. Model Validation

The model was internally validated using five-fold cross-validation. The performance of the new model was assessed using various metrics, including AUROC, Brier scores, and accuracy. The results of the model validation, specifically for in-hospital mortality, are presented in Supplementary Table S1 and Supplementary Figure S1. The model validation results showed good performance in terms of AUROC (>0.8), Brier scores, and accuracy for the primary outcome at different time points (3- and 6-month results not shown for brevity).

### 3.7. Comparative Subgroup Sensitivity Analysis of the Predictive Performance between the New SBP Score and the Original CTP, MELD, MELD-Na, iMELD, and MELD 3.0 Scores for 3-Month Mortality Prediction in Patients with HBV-Related Cirrhosis and SBP

**Figure 5:**
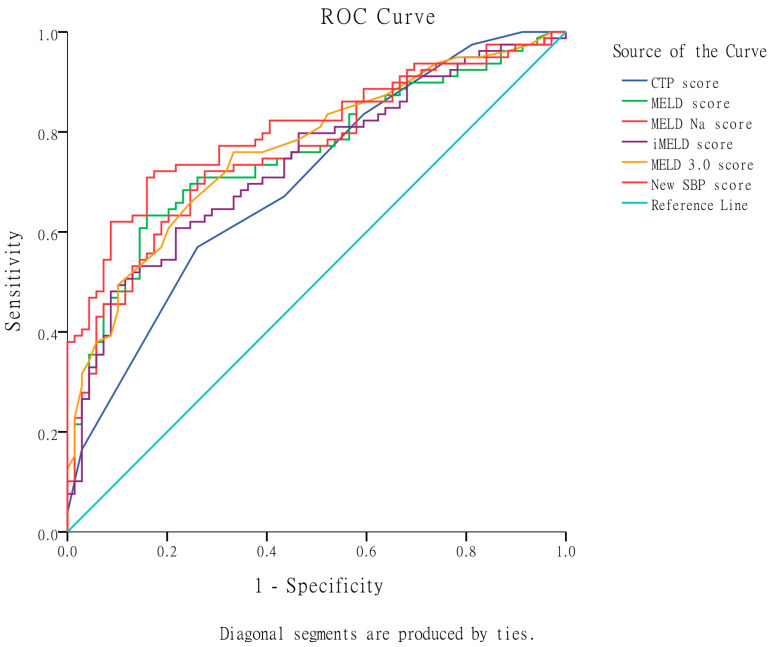
Comparison of the predictive performance of the original CTP, MELD, MELD-Na, iMELD, MELD 3.0, and the new SBP scores in predicting 3-month mortality in the HBV subgroup using AUROC. The new SBP score demonstrated significant superiority (0.813) over the CTP score, MELD, and iMELD scores. However, it did not show significant superiority over the MELD-Na and MELD 3.0 scores.

Given the high prevalence of HBV-related cirrhosis in Taiwan, a subgroup sensitivity analysis was conducted to assess the predictive accuracy of the scores in this specific patient population with SBP. In terms of 3-month mortality prediction, the new SBP score showed significant superiority over the CTP score, MELD, and iMELD scores, as indicated in Table 6 and Figure 5. However, it did not demonstrate significant superiority over the MELD-Na and MELD 3.0 scores.

### 3.8. The Cut-Off Values for the New SBP Score Model

The new SBP model demonstrated a cut-off value of −0.4697 for predicting in-hospital mortality, with mortality rates of 18.7% and 73.3% below and above this threshold, respectively (Supplementary Table S2). For 3-month mortality prediction, the new model’s cut-off value was 0.3712, with mortality rates of 33.6% and 84.9% below and above this value, respectively (Supplementary Table S3). Similarly, for 6-month mortality prediction, the cut-off value was 0.4690, corresponding to mortality rates of 40.8% and 83.6% below and above this threshold, respectively (Supplementary Table S4).

## 4. Discussion

SBP is a common and severe complication in cirrhotic patients with ascites [38], with a prevalence ranging from 10% to 30% in hospitalized patients [4,39]. Despite improvements in early recognition and effective antibiotic treatment, in-hospital mortality rates remain high, ranging from 20% to 40% [40,41]. Early identification of high-risk patients could potentially improve survival outcomes [42]. However, specific prediction models for SBP are currently unavailable. Therefore, our study aimed to compare the predictive ability of commonly used cirrhotic prediction models, including the CTP score and four MELD-based scores (MELD, MELD-Na, iMELD, MELD 3.0), for short-term mortality prediction (in-hospital, 3 and 6 month). Additionally, we sought to develop a novel model to enhance mortality prediction compared to conventional models. In this retrospective cohort study, we analyzed data from 327 cirrhotic patients who experienced their first SBP episode between January 2006 and August 2017. Clinical data were collected at the time of diagnosis, and patients were followed until March 2018 or death. Mortality prediction was assessed using AUROC analysis. HBV infection accounted for 45.3% of cirrhosis etiologies. In-hospital mortality was 39.4%, with cumulative 3-month and 6-month mortality rates of 51.4% and 60.9%, respectively. Among the existing prediction models, MELD 3.0 showed the highest AUROC in predicting in-hospital and 3-month mortality, surpassing iMELD, MELD, CTP, and MELD-Na. For 6-month mortality, iMELD showed the best performance. Our novel SBP model, incorporating age, serum Cr, total bilirubin, INR, PLT, sepsis, and HRS, demonstrated superior predictive accuracy for in-hospital mortality in all patients (AUROC 0.827) and 3-month mortality in HBV cirrhosis (AUROC 0.813), with HRS exhibiting the highest odds ratio. Internal validation and subgroup sensitivity analysis, explicitly focusing on HBV-related cirrhosis, further corroborated these findings. Our results emphasize the importance of developing a dedicated prognostic model for SBP and highlight the significance of preventing HRS development to improve the prognosis of SBP.

The CTP score has been extensively utilized for prognostic evaluation in cirrhotic patients for over four decades [43]. However, it possesses certain limitations, including the subjectivity of certain clinical parameters and its limited discriminative ability [44,45,46]. On the other hand, MELD has demonstrated superior accuracy in predicting 3-month survival compared to the CTP scoring system for cirrhotic patients awaiting liver transplantation in the United States [9]. Since February 2002, MELD has served as the primary reference system for organ allocation in liver transplantation in the United States [47]. Furthermore, MELD has shown effectiveness in predicting mortality across various chronic liver diseases and certain complications of portal hypertension, such as hepatic encephalopathy and acute variceal bleeding [48,49].

Several MELD-based models have been developed to enhance the accuracy of predicting wait-list mortality for liver transplantation. One such model is MELD-Na, which incorporates serum sodium (Na) as an additional predictor of wait-list mortality [50,51]. MELD-Na has been shown to accurately predict 6-month mortality in cirrhotic patients awaiting liver transplantation in a multicenter study [11]. Another model, the iMELD score, was developed to improve survival prediction in cirrhotic patients [12]. These MELD-based models have been compared and exhibited strong prognostic capabilities for outcome prediction in cirrhotic patients [16,17]. Recently, MELD has been updated to version 3.0 with the aim of reducing deaths while patients are on the liver transplantation waitlist [15]. However, there is limited information on the applicability of these models in the subgroup of patients with liver cirrhosis-related complications, particularly SBP, in regions with a high prevalence of chronic hepatitis B, such as Taiwan [22,52].

In our study, the MELD 3.0 score demonstrated significant superiority over the CTP score and non-significant superiority over MELD, MELD-Na, and iMELD scores in predicting in-hospital mortality. However, its AUROC of 0.786 did not reach the desired threshold of 0.8. To overcome this limitation, we developed a new SBP score model with an impressive AUROC of 0.827, surpassing the critical threshold of 0.8. The new SBP score model significantly outperformed the CTP score, MELD, MELD-Na, and iMELD scores, and slightly outperformed the MELD 3.0 score in predicting in-hospital mortality. For the 3-month mortality prediction, the new SBP score model demonstrated significant superiority over the CTP score and MELD, with an AUROC of 0.789. Subgroup sensitivity analysis focusing on HBV-related cirrhosis and SBP further improved the AUROC to 0.813. However, the performance of the new SBP score model in predicting 6-month mortality was not satisfactory, with an AUROC of 0.762, similar to that of the iMELD score. These findings suggest that the new SBP score model is particularly effective in predicting in-hospital and 3-month mortality, aligning with the current policy of assessing the risk of death within 90 days for liver transplants [53].

It is noteworthy that our study’s findings align with previous research emphasizing the detrimental impact of HRS on SBP prognosis. This is supported by the highest odds ratio in our SBP score model, highlighting the significant role of HRS in predicting SBP outcomes. These findings are consistent with a well-known study demonstrating the benefits of intravenous albumin treatment in conjunction with antibiotics, which reduces the incidence of renal impairment and mortality in cirrhotic patients with SBP, compared to antibiotic treatment alone [54]. This finding underscores the importance of preventing HRS development and highlights the potential value of interventions targeting renal function in improving SBP prognosis. 

The study has several limitations. Firstly, it is a retrospective study with a relatively small number of patients. Secondly, external validation was not conducted. Thirdly, being a single-center study, a multi-center study would be beneficial in the future. Despite these limitations, the findings underscore the need for a specific prognostic model for SBP and emphasize the importance of conducting a prospective and larger case series for validation.

## 5. Conclusions

In our study of cirrhotic patients with the first episode of SBP, the MELD 3.0 score outperformed the CTP score and showed a non-significant improvement compared to other MELD-based scores (MELD, MELD-Na, iMELD) in predicting in-hospital and 3-month mortality, although the AUROC values were below the desired threshold of 0.8. Our newly developed SBP model, incorporating age, serum Cr, total bilirubin, INR, PLT, sepsis, and HRS, achieved an impressive AUROC value exceeding 0.8 for in-hospital mortality in all patients and 3-month mortality in HBV cirrhosis. Internal validation and subgroup sensitivity analysis focusing on HBV-related cirrhosis supported these findings. These results highlight the need for a specific prognostic model for SBP and underscored the importance of preventing HRS development to improve SBP prognosis.

## Figures and Tables

**Table 1:** Demographic and baseline clinical characteristics of 327 patients with the first episode of SBP.

Baseline Parameters	Values
Clinical Parameters	
Age, mean ± SD	57.1 ± 13.6
Male No. (%)	236 (72%)
Etiology No. (%)	
Alcohol	90 (27.5%)
HBV	148 (45.3%)
HCV	78 (23.9%)
Others	11 (3.4%)
Blood culture positive	42(12.8%)
Hepatorenal syndrome	31(9.5%)
Hepatic encephalopathy	
Grade 0	243(74.3%)
Grade 1	29(8.9%)
Grade 2	32(9.8%)
Grade 3	15(4.6%)
Grade 4	8(2.4%)
Ascites	
Mild	43(13.2%)
Moderate	76(23.2%)
Severe	208(63.6%)
**Laboratory Parameters** **Median (IQR)**	
Creatinine (mg/dL)	1.2 (0.86–2.41)
Bilirubin Total (mg/dL)	4.1 (1.9–9.8)
Sodium (mEq/L)	135 (131–139)
Albumin (g/dL)	2.4 (2.2–2.8)
INR	1.64 (1.39–2.20)
WBC (10^3^/μL)	7.9 (5.20–12.80)
Hemoglobin (g/dL)	9.6 (8.4–11.0)
PLT (10^3^/μL)	74.0 (48.0–122.0)
Ascites PMN (cells/mm^3^)	1393.5 (344.3–4203.0)
CTP score	10.1 ± 1.9
MELD score	22.7 ± 9.3
MELD-Na score	25.1 ± 8.5
iMELD score	45.6 ± 10.4
MELD 3.0 score	25.4 ± 8.5

**Table 2:** Statistical differences in scores among CTP and four MELD-based models for predicting in-hospital, 3-month, and 6-month mortalities between mortality and non-mortality groups.

	Mortality	Non-Mortality	*p*
In-hospital	*n* = 129 (39.4%)	*n* = 198 (60.6%)	
CTP score	10.95 ± 1.785	9.58 ± 1.839	<0.001
MELD score	28.3977 ± 10.28562	19.0414 ± 6.37866	<0.001
MELD-Na score	30.3214 ± 8.59616	21.7216 ± 6.57153	<0.001
iMELD score	51.8581 ± 10.17405	41.4468 ± 8.26332	<0.001
MELD 3.0 score	30.6357 ± 8.50546	21.9747 ± 6.52013	<0.001
3-Month	*n* = 168 (51.4%)	*n* = 159 (48.6%)	
CTP score	10.64 ± 1.865	9.57 ± 1.861	<0.001
MELD score	26.5179 ± 10.19027	18.7327 ± 6.21459	<0.001
MELD-Na score	28.7469 ± 8.74900	21.2759 ± 6.36454	<0.001
iMELD score	50.0182 ± 10.50086	40.8371 ± 790264	<0.001
MELD 3.0 score	29.0714 ± 8.70169	21.5031 ± 6.24892	<0.001
6-Month	*n* = 199 (60.9%)	*n* = 128 (39.1%)	
CTP score	10.50 ± 1.864	9.52 ± 1.903	<0.001
MELD score	25.5815 ± 9.90270	18.3030 ± 6.18074	<0.001
MELD-Na score	27.8953 ± 8.7364	20.7905 ± 6.43326	<0.001
iMELD score	49.1069 ± 10.34567	40.0304 ± 7.71442	<0.001
MELD 3.0 score	28.1457 ± 8.52293	21.1094 ± 6.43224	<0.001

*n* = number of patients.

**Table 3:** Comparison of the predicting ability of the original CTP, MELD, MELD-Na, iMELD, MELD 3.0, and the new SBP scores in predicting in-hospital mortality by AUROC and DeLong test.

Predicting In-Hospital Mortality						
	CTP Score	MELD Score	MELD-Na Score	iMELD Score	MELD 3.0 Score	New SBP Score
AUROC	0.701	0.775	0.783	0.780	0.786	0.827
(95% CI)	0.64–0.76	0.72–0.83	0.73–0.83	0.73–0.83	0.73–0.84	0.78–0.87
*p*-value	<0.001	<0.001	<0.001	<0.001	<0.001	<0.001
CTP score						
MELD score	0.0013					
MELD-Na score	0.0003	0.4608				
iMELD score	0.0041	0.7843	0.8778			
MELD 3.0 score	0.0001	0.2789	0.5814	0.7370		
New SBP score	<0.0001	0.0196	0.0471	0.0240	0.0651	

**Table 4:** Comparison of the predicting ability of the original CTP, MELD, MELD-Na, iMELD, MELD 3.0, and the new SBP scores in predicting 3-month mortality by AUROC and DeLong test.

Predicting 3-Month Mortality						
	CTP Score	MELD Score	MELD-Na Score	iMELD Score	MELD 3.0 Score	New SBP Score
AUROC	0.656	0.737	0.755	0.753	0.760	0.789
(95% CI)	0.60–0.71	0.68–0.79	0.70–0.81	0.70–0.81	0.71–0.81	0.74–0.84
*p*-value	<0.001	<0.001	<0.001	<0.001	<0.001	<0.001
CTP score						
MELD score	0.0007					
MELD-Na score	<0.0001	0.0799				
iMELD score	0.0007	0.4011	0.8833			
MELD 3.0 score	<0.0001	0.0209	0.4002	0.6702		
New SBP score	<0.0001	0.0266	0.1499	0.1074	0.2160	

**Table 5:** Comparison of the predicting ability of the original CTP, MELD, MELD-Na, iMELD, MELD 3.0, and the new SBP scores in predicting 6-month mortality by AUROC and DeLong test.

Predicting 6-Month Mortality						
	CTP Score	MELD Score	MELD-Na Score	iMELD Score	MELD 3.0 Score	New SBP Score
AUROC	0.640	0.728	0.745	0.752	0.742	0.762
(95% CI)	0.58–0.70	0.67–0.78	0.69–0.80	0.70–0.80	0.69–0.80	0.71–0.81
*p*-value	<0.001	<0.001	<0.001	<0.001	<0.001	<0.001
CTP score						
MELD score	0.0007					
MELD-Na score	<0.0001	0.0623				
iMELD score	0.0001	0.1345	0.5149			
MELD 3.0 score	<0.0001	0.0772	0.8966	0.4950		
New SBP score	0.0008	0.1115	0.4330	0.6602	0.4094	

**Table 6:** Comparison of the predicting ability of the original CTP, MELD, MELD-Na, iMELD, MELD 3.0, and the new SBP scores in predicting 3-month mortality in the HBV-related cirrhosis subgroup.

Predicting 3-Month Mortality in HBV Subgroup						
	CTP Score	MELD Score	MELD-Na Score	iMELD Score	MELD 3.0 Score	New SBP Score
AUC	0.700	0.755	0.760	0.736	0.765	0.813
(95% CI)	0.62–0.78	0.68–0.83	0.68–0.84	0.66–0.82	069–0.84	0.73–0.89
*p*-value	<0.001	<0.001	<0.001	<0.001	<0.001	<0.001
CTP score						
MELD score	0.1442					
MELD-Na score	0.1014	0.6895				
iMELD score	0.4245	0.5233	0.2928			
MELD 3.0 score	0.0711	0.4827	0.5569	0.2254		
New SBP score	0.0291	0.0476	0.0886	0.0446	0.2035	

## Data Availability

Data and study materials will be made available to other researchers upon request via email, along with their IRB approval document.

## Supplementary Materials

The following supporting information can be downloaded at: https://www.mdpi.com/article/10.3390/diagnostics13152578/s1. Table S1: Model internal validation by 5-fold cross-validation. Table S2: The cut-off values for the new SBP score model in predicting in-hospital mortality. Table S3: The cut-off values for the new SBP score model in predicting 3-month mortality. Table S4: The cut-off values for the new SBP score model in predicting 6-month mortality. Figure S1: The AUROC curve of the training set and the test (validation) set for predicting in-hospital mortality.

## Abbreviations

ALT	alanine aminotransferase
AST	aspartate aminotransferase
AUROC	area under the receiver operating characteristic curve
GI	gastrointestinal
HCC	hepatocellular carcinoma
HCV	hepatitis C virus
HRS	hepatorenal syndrome
LC	liver cirrhosis
PLT	platelet
PMN	polymorphonuclear neutrophils
PPV	positive predictive value
PT-INR	Prothrombin time–International Normalized Ratio
SBP	spontaneous bacterial peritonitis
WBC	white blood cell
