# Supplementary material for: A Validated Composite Score Demonstrates Potential Superiority to MELD-Based Systems in Predicting Short-Term Survival in Patients with Liver Cirrhosis and Spontaneous Bacterial Peritonitis—A Preliminary Study
Source: Diagnostics (Basel). 2023 Aug 2;13(15):2578. doi: 10.3390/diagnostics13152578 (PMC10417459; doi:10.3390/diagnostics13152578)
Supplement: Supplementary file 1 [file diagnostics-13-02578-s001.zip › diagnostics-2480804-supplementary.pdf]

Supplementary Table S1. Model internal validation by 5-fold cross-validation

Predicting in-hospital mortality						
**Dataset**	**Total N**	**AIC**	**R-Square**	**RMSE**	**AUROC**	**Brier Score**
**Train**	261	275.2	0.2566665	0.4178324	0.8148581	0.263008
**Test**	66	63.72	0.3990093	0.4047687	0.8365385	0.4610844
**accuracy = 0.7424242**						

Supplementary Table S2. The cut-off values for the new SBP score model in predicting in-hospital mortality

Mortality above or below the cut-off values	
In-hospital, n=129 (39.4%)	
In-hospital mortality new SBP score	Mortality rate
≧-0.4697	73.38709677%
<-0.4697	18.711921182%

Supplementary Table S3. The cut-off values for the new SBP score model in predicting 3-month mortality

Mortality above or below the cut-off values	
Three-month, n=168 (51.4%)	
Three-month mortality new SBP score	Mortality rate
≧ 0.3712	84.95575221%
< 0.3712	33.64485981%

Supplementary Table S4. The cut-off values for the new SBP score model in predicting 6-month mortality

Mortality above or below the cut-off values	
One month, n=199 (60.9%)	
Six-month mortality new SBP score	Mortality rate
≧ 0.4690	83.66013072%
< 0.4690	40.8045977%

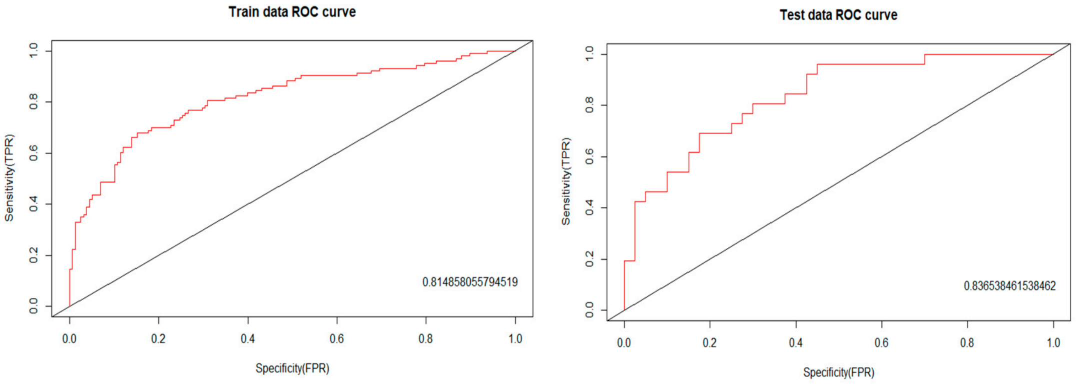

Supplementary Figure S1. The AUROC curve of the training set and the test (validation) set for predicting in-hospital mortality. The AUROC of the training cohort for the new SBP score was 0.815, and for the validation cohort, it was 0.837, both> 0.8.
